# What do the US advanced kidney disease patients want? Comprehensive pre-ESRD Patient Education (CPE) and choice of dialysis modality

**DOI:** 10.1371/journal.pone.0215091

**Published:** 2019-04-09

**Authors:** Ashutosh M. Shukla, Colin Hinkamp, Emma Segal, Tezcan Ozrazgat Baslanti, Teri Martinez, Michelle Thomas, Ramya Ramamoorthy, Shahab Bozorgmehri

**Affiliations:** 1 Department of Medicine, North Florida / South Georgia Veteran Healthcare System, Gainesville, Florida, United States of America; 2 Division of Nephrology, Hypertension, and Transplantation, Department of Medicine, University of Florida, Gainesville, Florida, United States of America; 3 Dialysis Clinic Inc (DCI), Gainesville, Florida, United States of America; 4 Department of Medical Socidal Worker, UF Health, Gainesville, Florida, United States of America; Peking University First Hospital, CHINA

## Abstract

Improvement in Home Dialysis (HoD) utilizations as a mean to improve the patient reported and health services outcomes, has been a long-held goal of the providers and healthcare system in United States. However, measures to improve HoD rates have yielded limited success so far. Lack of patient awareness of chronic kidney disease (CKD) and its management options, is one of the important barriers against patient adoption of HoD. Despite ample evidence that Comprehensive pre-ESERD Patient Education (CPE) improves patient awareness and informed HoD choice, use of CPE among US advanced CKD patients is low. Need for significant resources, lack of validated data showing unequivocal and reproducible benefits, and the lack of validated CPE protocols proven to have consistent efficacy in improving not only patient awareness but also HoD rates in US population, are major limitations deterring adoption of CPE in routine clinical practice. We recently demonstrated that if a structured, protocol based CPE is integrated within the routine nephrology care for patients with advanced CKD, it substantially improves informed HoD choice and utilizations. However, this requires establishing CPE resources within each nephrology practice. Efficacy of a stand-alone CPE model, independent of clinical care, has not been examined till date. In this report we report the efficacy of our structured CPE protocol, delivered outside the realm of routine nephrology care—as a stand-alone patient education program, in a geographically distant region, and show that: when provided opportunity for informed dialysis choice, a majority of advanced CKD patients in US would prefer HoD. We also show that initiating CPE leads to accelerated growth in HoD utilizations and reduces disparities in HoD utilizations, goals for system improvements. Finally, the reproducibility of our structured CPE protocol with consistent efficacy data suggest that initiating such programs at institutional levels has the potential to improve informed dialysis selection and HoD rates across any similar large healthcare institute within US.

## Introduction

Home dialysis (HoD) is grossly underutilized for the management of end stage renal disease (ESRD) in US. Due to equivalence in survivals, and trends for better patient-oriented and health services outcomes, i.e. health related quality of life, patient satisfaction, cost of care, etc., major ESRD stakeholders including physicians, payors and policy makers, have advocated increasing the HoD use in US.[1–6] Despite these agreements the growth of HoD in US over last decade has been slow.[7]

Selection of HoD by advanced CKD patients requires comprehension of various forms of dialysis therapy and their impact on quality of life. Thus, patients’ lack of awareness of CKD and its management options including available dialysis therapies can significantly hinder selection of HoD. Recently a number of investigators have shown that patients’ awareness of CKD and its management options continue to be suboptimal, even in the current era.[5, 8, 9] In view of these, major professional renal societies as well as Center for Medicare and Medicaid Studies (CMS) recommend that all patients with advanced CKD should be provided with Comprehensive pre-ESRD Education (CPE) to facilitate informed dialysis choice.[10–13] Reports from Canada and Europe have shown that CPE not only improves quality of CKD care and patient awareness, but also increases informed HoD choice,[5] with nearly half of those receiving CPE preferring HoD.[3, 6, 14–17] Unfortunately, due to multiple patient, provider and system based limitations, which include lack of validated CPE protocols, lack of consistent outcome data in US patients, and the need for resources among others; few providers in US provide CPE and opportunity for informed dialysis choice for their advanced CKD patients.[18] The end-result is that pre-ESRD education is provided infrequently, and when provided, is done disparately among those with socio-economic advantage leading to limited and disparate HoD use. Need for effective CPE protocols that can be reproduced and universalized, and its universal non-selective application has been repeatedly emphasized. [19]

We have developed a concise CPE protocol, and recently showed that our concise protocol when interwoven within the ongoing clinical nephrology care within a university network, can substantially improve informed HoD choice and use, and increase HoD prevalence. [20] However, this requires establishing CPE resources within each nephrology practice. Efficacy of a stand-alone, educational model of CPE that can be disseminated across multiple practices has not been tested for US patients. The current paper aims to highlight 1) The patient behavior and preference characteristics with reference to a stand-alone CPE program, while receiving ongoing CKD care through their primary nephrologist, 2) its effects on the rates of selection and use of HoD among advanced CKD patients, and 3) the reproducibility of our CPE protocol across a different geographical area within US.

## Methods

This is a retrospective-prospective analysis of the first 18 months of a newly established CPE clinic at University of Florida (UF). Once enrolled the patients were followed prospectively for their need, and modality of dialysis. The data were extracted from the UF Health electronic medical records after the appropriate approvals from the IRB 01, University of Florida Institutional Review Board, Gainesville Health Science Center.

Based on the success of our prior published CPE protocol, we established a comprehensive pre-ESRD education (CPE) clinic at UF Health with an aim to improve patient awareness, promote informed dialysis choice, and examine its impact on the rates of selection and use of HoD modalities. In contrast to our prior experience, we separated the CKD care form CPE such that, the CPE clinic was geographically separated from the nephrology care clinics, and only provided kidney disease education. Patients continued their nephrology care with their chronic providers. Further in order to eliminate a selection bias, we adopted a universal CPE referral and access for all patients with stage 4 & 5 CKD followed at university of Florida, irrespective of their socio-economic and literacy background. Details of the CPE protocol are available in supplement.

### Brief CPE protocol

All patients with advanced (stage 4 and 5) CKD followed at the UF renal clinics were advised to undergo CPE (S1 File). Patients who refused CPE and those who ‘no showed’ for a minimum of 3 times for their self-chosen CPE appointments were aggregated as ‘No Shows.’ Patients were encouraged to attend CPE sessions with their caregivers or family members when applicable. All patients visiting the CPE clinic underwent a detailed interactive classroom education for kidney disease of a minimum of one hour duration. The classroom sessions were held in a group and interactive format, followed the topics usually recommended by the National Kidney Foundation’s patient education syllabus, and detailed information about kidneys’ structure, function, stages of CKD, its symptoms, management and mitigating strategies,and management options for ESRD which included renal transplantation, conservative care and various types of renal replacement therapies (RRT). Classroom sessions were followed by a face-to-face individual counseling where, all information provided thorugh the classroom session were reviewed and concerns addressed. Final process for CPE involved a *Life-Simulation on Dialysis* discussion, which involved a virtual walk through the patients’ weekly life on dialysis. Post-CPE, dialysis choice were recorded in an active manner, i.e. patients were asked to choose a dialysis modality and once chosen provide rationale for their choice.’ Those uncertain of the dialysis modality were asked to attend additional sessions.

### Statistical analysis

Demographics and clinical characteristics were examined using one-way analysis of variance (ANOVA) and Kruskal-Wallis tests for normally and non-normally distributed continuous variables, respectively, and chi-square or Fisher’s exact tests for categorical variables. We used univariate and multivariable logistic regression (MLR) models to determine the association between demographics and clinical factors and the individual choice of HoD. We included age, gender, race, insurance status, marital status, smoking status, body mass index, estimated glomerular filtration rate (eGFR), congestive heart failure and diabetes mellitus *a priori* in the MLR model. All significance tests were two-sided, with a p value < 0.05 considered statistically significant. Statistical analyses were performed using Statistical Analysis Software (SAS) version 9.4 (SAS Institute Inc, Cary, North Carolina).

## Results

Table 1 shows the demographic distribution of the entire cohort with reference to their dialysis modality selections.

**Table 1:** Demographic and baseline statistics of patients by their HoD choice as well as eventual use.

	Dialysis Choice				Dialysis Use	
	HoD Choice	IHD Choice	Conservative Choice	Undecided	HoD use	IHD use
Age	58 ± 14	60 ± 14	63 ± 4.2	62 ±13	55 ± 16	59 ± 14
MDRD eGFR	16 ± 10	16 ± 8	18 ± 5.4	16 ± 6	12 ± 5	12 ± 4
BMI	31 ± 8	31 ± 6	31 ± 5	33 ± 8	30 ± 6	31 ± 8
No. of CPE visits	1.2 ± 0.4	1.3 ± 0.5	1.2 ± 0.4	1.3 ± 0.6	1.3 ± 0.5	1.2 ± 0.4
Female Gender	44%	37%	30%	20%	37%	52%
Non-white Race	37%	47%	48%	20%	34%	40%
Non-private insurance	58%	88%	75%	40%	58%	71%
Living alone	42%	53%	50%	40%	47%	40%
Diabetes	53%	58%	45%	60%	41%	60%
CHF	18%	11%	25%	20%	20%	20%

Age (years), MDRD eGFR (ml/min), BMI (kg/m^2^) and no. of CPE visits are reported in Mean ± standard deviation; All non-private insurance, e.g. Medicare, Medicaid and Veteran Administration based insurance, were grouped together for analysis

### CPE participation

**Fig 1:**
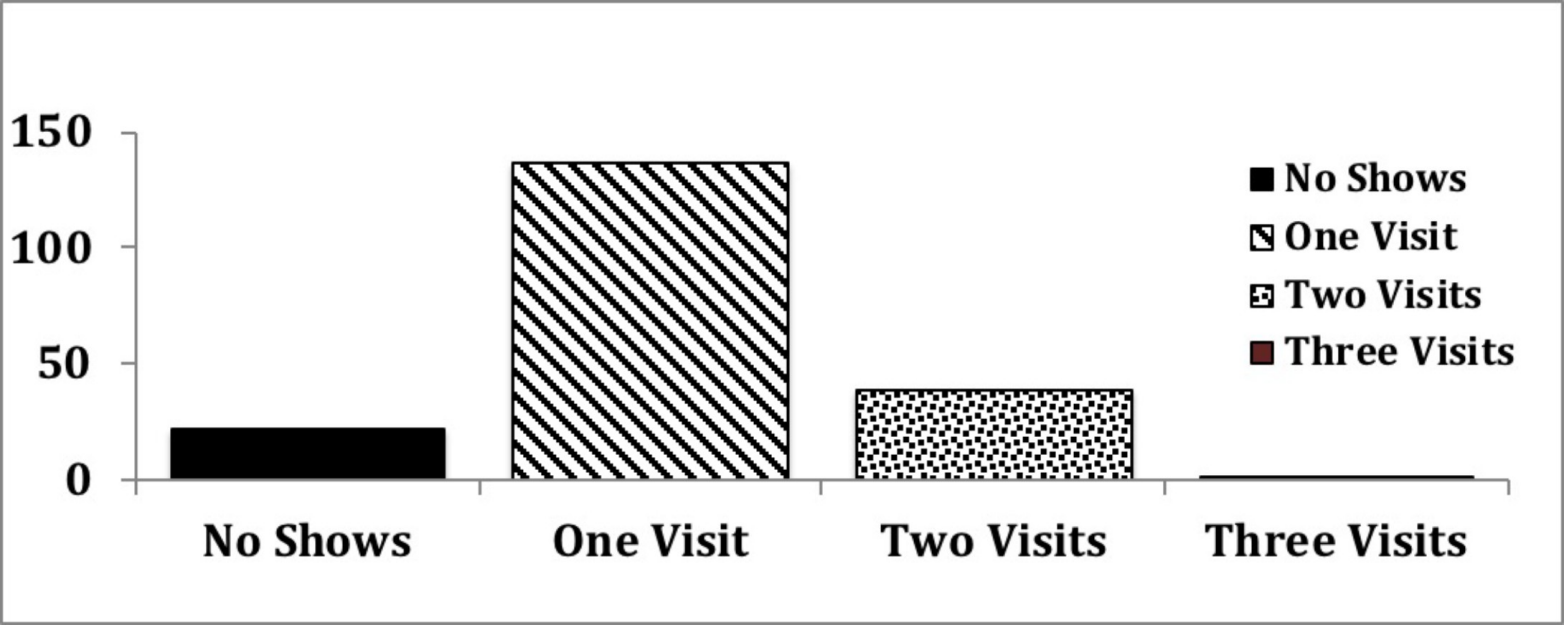
Dialysis modality choice by the number of CPE clinic visit. Fig 1 shows the number of CPE clinic patients elected to attend, when provided with an option to decide a follow up visit based on their comfort level for reaching dialysis choice. Each column is further subdivided into the patient choice for the different dialysis modalities with bars representing the proportion of patients reaching the given modality choice.

A total of 245 patients were identified to have stage 4/5 non-dialysis CKD within UF nephrology clinics, and referred for CPE over the initial 18 month period. Sixty eight (28%) patients who were referred to and offered CPE, chose not to participate in the program and were referred to as ‘No Shows’ cumulatively. Of the 177 patients enrolled, 136(77%) chose to participate in only one session of CPE whereas 39 (22%) and 2 (1%) attended 2 and 3 sessions respectively (Fig 1). Over the following 34 months follow up, no patient chose to attend more than 3 sessions of CPE.

### Dialysis choice

**Fig 2:**
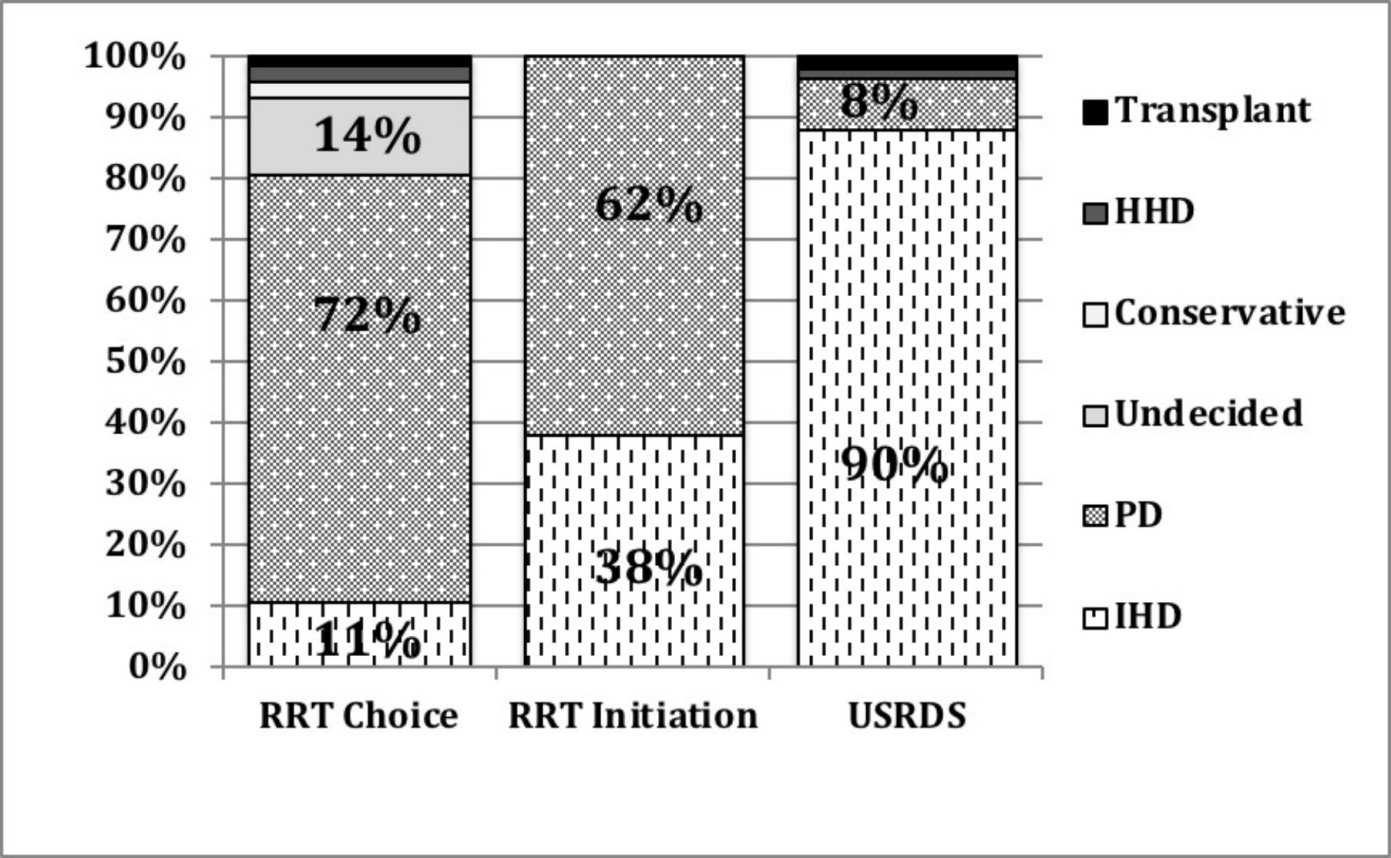
Impact of CPE on the choice and use of HoD compared to the prevalent USRDS data. Fig 2 shows the impact of CPE on the selection and use of various renal replacement therapies compared to the concurrent prevalent USRDS data. [27] HHD: Home hemodialysis; PD: peritoneal dialysis; IHD: In-center hemodialysis; RRT: Renal replacement Therapy.

Among the 177 patients who attended the CPE, 129 (75%) chose HoD modalities with home peritoneal dialysis (PD) selected by 124 patients (70%) and home HD (HHD) selected by 5 (2.8%). In sharp contrast to the national incident ESRD statistics, provided only for illustrative comparison, in-center hemodialyisis (IHD) was chosen by only 19 patients (10.7%) and 24 patients (13.5%) remained undecided, including 2 patients opting only for transplantation (Fig 2). The number of clinic visits and the stage of CKD (4 Vs. 5) had no significant impact on the modality of dialysis chosen. When accounted for the whole population, i.e. including those who were identified and preferred for CPE, but did not to attend CPE, 52% of overall advanced CKD population chose HoD therapy. Univariate and multivariate analyses showed that most socio-demographic and comorbidity variables had no impact on individual choice of HoD, apart from the type of insurance where Medicare/Medicaid recepients were less likely to choose HoD compared to those with private insurance (Table 2).

**Table 2:** Univariate and multivariable regression analysis of factors impacting the HoD choice.

		Univariate model		Multivariable model	
	Referent	Odds Ratio (95%CI)	p value	Odds Ratio (95%CI)	p value
Age		1.00 (0.96–1.04)	0.902	1.03 (0.97–1.10)	0.270
BMI		1.00 (0.93–1.08)	0.977	1.02 (0.92–1.15)	0.680
MDRD eGFR		1.00 (0.94–1.06)	0.974	1.01 (0.94–1.08)	0.875
Gender, female	Male	1.31 (0.43–4.02)	0.638	2.53 (0.57–11.25)	0.222
Race, nonwhite	White	0.66 (0.22–2.00)	0.460	0.57 (0.10–3.26)	0.527
Non-private insurance	Private insurance/self-pay	0.22 (0.05–1.02)	0.054	0.07 (0.01–0.76)	0.029
Marital status, married/living with a partner	Living alone	1.82 (0.59–5.58)	0.298	1.92 (0.32–11.43)	0.475
Smoking status, current/past smoker	Never smoker	0.72 (0.15–3.44)	0.675	1.21 (0.11–12.82)	0.877
Diabetes mellitus		0.74 (0.24–2.26)	0.593	0.43 (0.07–2.60)	0.356
Congestive heart failure		1.48 (0.31–7.12)	0.622		1.24 (0.20–7.77)	0.815

Age is referenced in years, MDRD eGFR in ml/min, and BMI is referenced in kg/m^2^.

### Dialysis initiation

**Fig 3:**
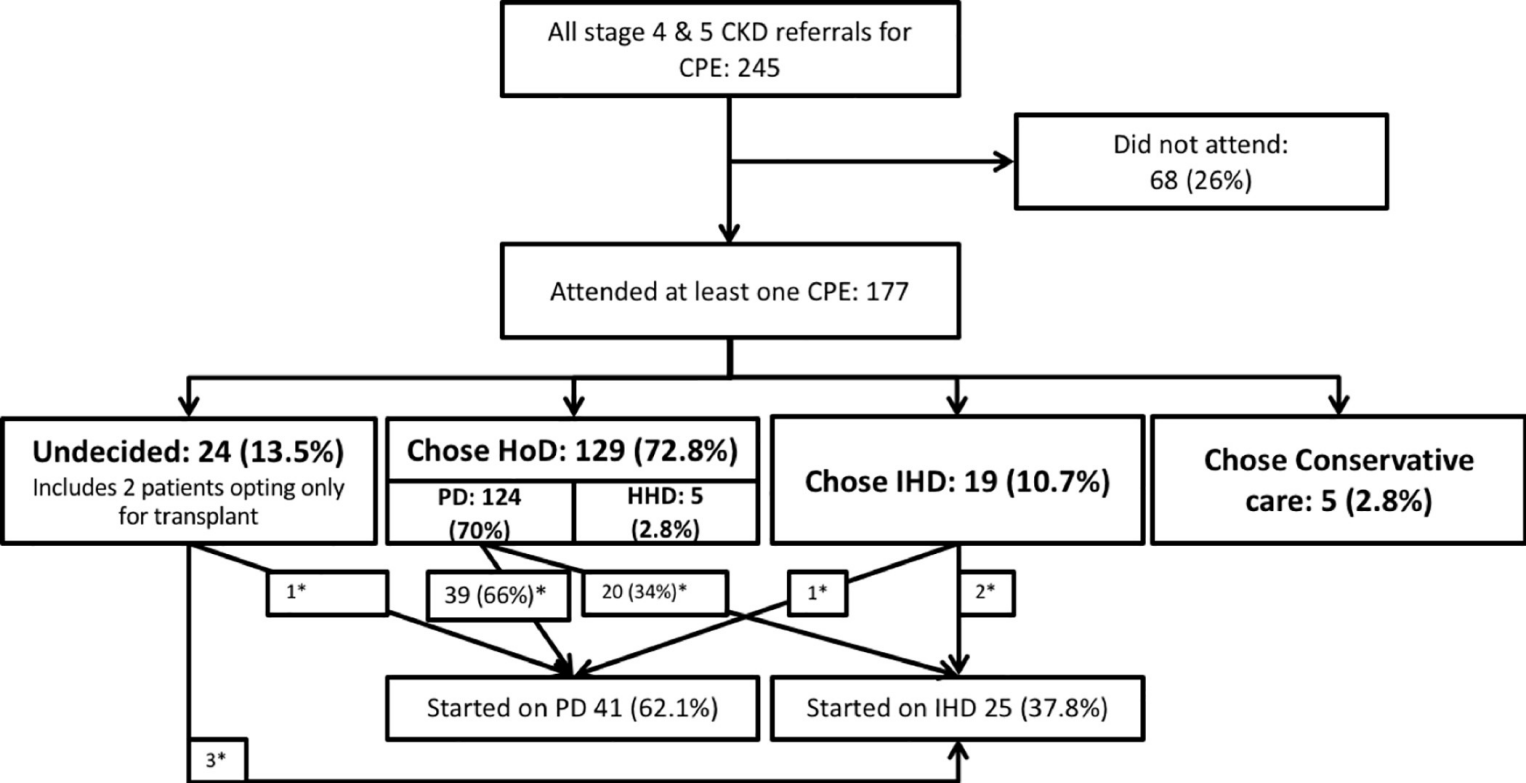
Flow-chart showing patient attendance patterns in a universal CPE program. * Reflects the no. of patients (%) who initiated renal replacement therapies at the end of the follow up period; CPE: Comprehensive Pre-ESRD patient Education; HoD: Any form of home dialysis; IHD: In-Center Hemodialysis.

Sixty six (37.2%) of patients who attended CPE required initiation of RRT. Amongst these, 41 (62.1%) initiated HoD, and 25 (37.8%) initiated IHD (Fig 3). Among 59 of the incident ESRD patients who initially chose HoD, 39 (66%) initiated HoD therapies whereas the remaining third initiated IHD. All patients initiated on IHD had the initial choice of IHD for their RRTs.

### HoD growth

**Fig 4:**
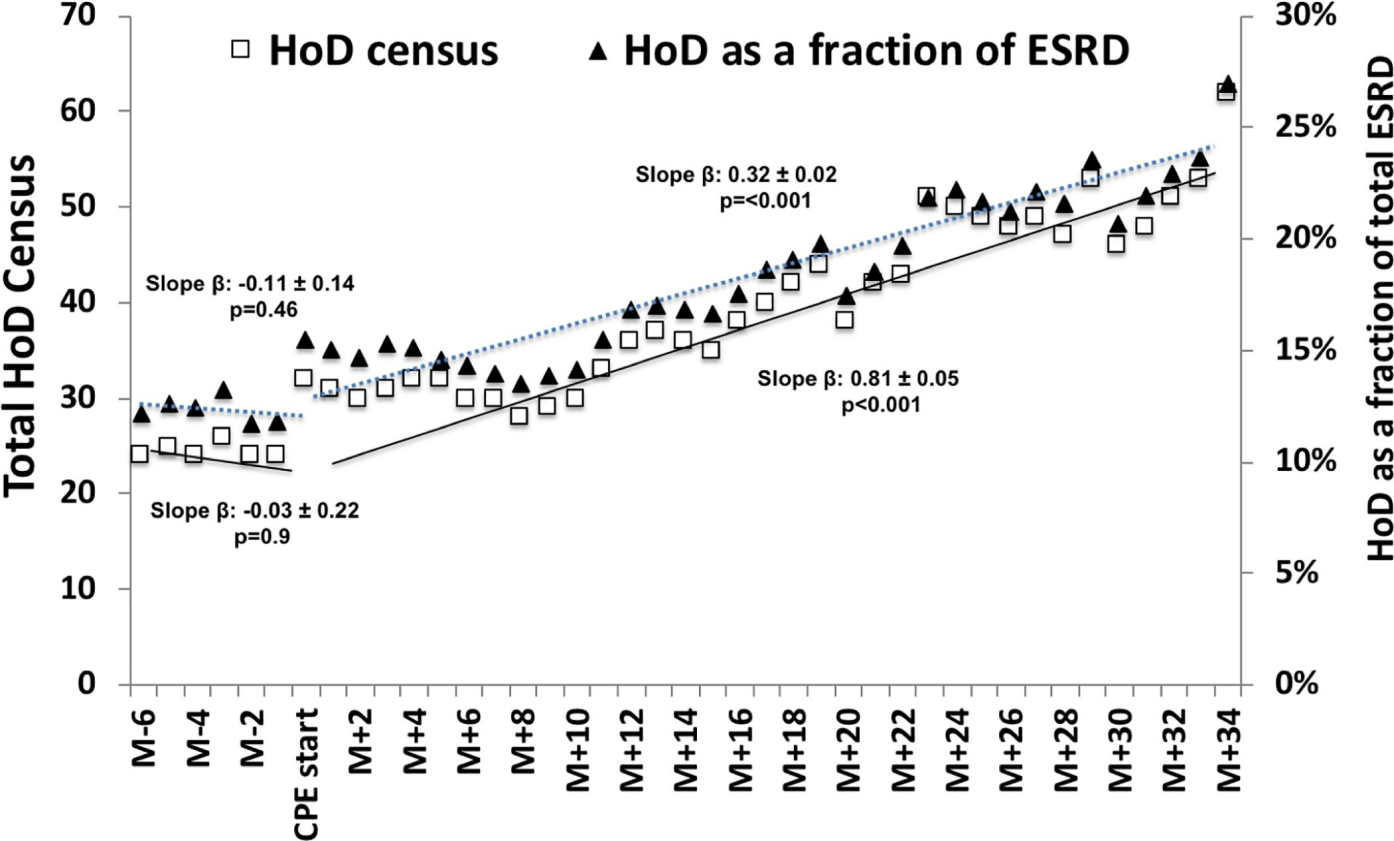
Growth of HoD utilization before and after the initiation of CPE program. Fig 4 represents the HoD utilization before and after the initiation of CPE program. The choice and utilization data in the text represents the initial 18 months study period for which the data is available. The extended 34 month graph is meant to represent the ongoing impact of CPE program on the HoD growth beyond the study period.

Fig 4 shows the trends of HoD census within the university nephrology practices before and upto 34 months after the initiation of the CPE program. Initiation of CPE program resulted in 183% growth in the HoD census within the university nephrology practice over the study period and continued to expand during the study analysis period at a linear rate. At the end of 34 months post-CPE initiation, HoD prevalence increased to 27% of all ESRD patient population compared to an average of 12% prevalence for the same centers in the prior decade (Fig 4).

## Discussion

Despite near universal agreements among various ESRD stakeholders and a number of position papers and policy changes targeting greater informed HoD utilizations, growth of HoD in US has been slow and difficult to achieve.[7] It is well established through numerous international studies that CPE, in addition to improving many aspects of CKD care, improves patient awareness and increases informed HoD choice with reported rates for HoD, over 50% among those receiving the education.[3, 6, 14–16] Yet, few in US provide CPE, universally to all of their advanced CKD patients. A recent USRDS analysis of CMS billing database showed that Kidney Disease Education services are billed in less than 2% of the eligible population. [21]

Lack of consistent efficacy data establishing the benefits of CPE for US CKD patients and lack of resources and expertise to provide CPE are significant factors for this wide-spread underuse of CPE.[18] Two prominent papers examining the efficacy of CPE are available from US. In one report examining the efficacy of an university CPE program, where referral was at the discretion of the provider, Liebman et al. enrolled 217 patients over a 5 years period. They showed that, in this pre-selected group, nearly half (49%) of those provided with CPE selected HoD but, only a third (30%) eventually used HoD.[22] In the second study reporting the results of a nationwide quality improvement program for a large dialysis organization, Lacson et al. showed modality education led to HoD selection in nearly quarter of the patients (24%). Unfortunately, only few practices participated in this project, and it was unclear whether all patients within those practices participated. [16] Selection bias appears to have a major role in this report as the patients who received CPE had greater prevalence of characteristics commonly associated with HoD use, and a much larger (9 times larger) ‘control cohort’ had substantially lower (3.7%) HoD rates, yielding the overall HoD rates of 5.9%; comparable to then prevalent national statistics. Compared to these, in a first of its kind report, we recently showed that our model of CPE with lifestyle simulation discussions can result in HoD selection rates for US CKD patients which are comparable to the published international reports. We further showed that initiating a CPE program can result in rapid rise in prevalence of HoD within a healthcare infrastructure, with our study showing 216% growth in HoD census to reach a 38% HoD prevalence.

The current study furthers these findings in several important manners. First, a unique aspect of our study is how it deals with the selection bias evident in prior published US studies. The referral to CPE in our cohort was not left to the individual providers (to minimize the selection bias) but all patients within the university renal clinic were protocolized to be referred to the CPE clinic. In this manner, the study provides a unique insight into the overall population preference patterns for the CPE process. CMS recommends informed dialysis choice for all patients transitioning to ESRD and has establish a policy to provide ‘limited’ reimbursement for upto six Kidney Disease Education (KDE) sessions.[4] The rationale for such distributive and likely sub-optimal payment structure is unknown. Our prior study showed that even when mandated for their care only half the patients prefer coming for a second session of the CPE and the CPE attendance beyond 3 sessions is uncommon. [20] The current findings show the patient attendance pattern for CPE, if CPE is not mandated for their CKD care. About quarter of the patients did not attend any CPE despite the protocol for uniform CPE and repetitive efforts. Though we cannot objectively comment on the reasons for non-attendance, we presume that the concerns of limitations in health literacy, disease denial, or fear etc. may play important role. Of those who attended CPE, three quarters attended only one session and the attendance for the third session was only about 2%. Since we protocolized our CPE attendance based on their expected time to ESRD, these patient attendance patterns give an indirect indications for patient preferences for CPE. Lack of appropriate reimbursement has been argued as one of the important factors limiting universal CPE in US. This patient preference pattern combined with the need for extended duration of CPE sessions when the patients do attend, argue for considerations for an alternate time-based reimbursement approach. This may allow for more community practices to adopt universal CPE.

Second, our study provides an insight into the preference patterns of US advanced CKD population for HoD. Fadem et al. showed that among the 56% of the ESRD who knew about the existence of HoD, 10% chose HoD.[23] Lacson et al. showed that in a national education program, 24% chose HoD, and Liebman et al. showed HoD preferences at 50% but use at 30%. These studies suffer from a combination of selection bias and lack of details of protocol concerns, and their impact on HoD utilization (prevalence) rates are unknown.[16] Surveys of the practicing nephrologists have indicated that HoD may be appropriate for about 20–40% of prevalent ESRD population.[1–3] At the same time if hypothetically needed for themselves, over 90% would prefer some form of HoD.[24] True informed estimates of these preferences for US patients are not available. Due to a universal approach to CPE, we are able to comment on these statistics better. We for the first time show that even for US patients, three quarters of those provided with CPE will choose CPE, and starting a universal CPE program leads to over 50% HoD choice among prevalent advanced CKD patients, even after accounting those who may not choose to participate in CPE. Such high rates of patient preference pattern are a strong rationale for initiating CPE program at each individual institutes.

Third, we show a patient-oriented method of improving the HoD prevalence rates. The published US reports show that the provision of CPE has substantial selection bias, i.e. higher rates of CPE and thus HoD among the socio-economically privileged patients.[19] [25] Unfortunately, these strategy has led to limited growths in overall HoD pravelence. We employed an ‘all-comers’ strategy, offering CPE irrespective of the socio-economic and educational status, and showed that despite this, nearly three quarters of them chose HoD, leading to rapid and substantial growth in HoD prevalence at our institutes. Mehrotra et al. recently in a USRDS analysis showed disparate HoD use and greater HoD technique failure among minorities.[25] Our analysis shows that a patient-centered CPE can eliminate the socio-demographic barriers, commonly seen in cohort-based reports, and that this ‘all-comers’ strategy may be important if a true growths in HoD rates are to be visualized.

Fourth, our results validate the portability of our CPE protocol. Though major national organizations i.e. ASN, NKF, VA etc. have provided an outline for CPE, a validated protocol with replicable results is not available. Experts have emphasized the need for such a validated approach. [19] The results of this study are highly congruent to our prior published study using the same CPE protocol, with HoD selection rates of 70% and 74%, and rapidly rising HoD prevalence rates over 18 months. [20] We believe this provides evidence that with appropriate training, our concise CPE protocol can be implemented across any similar large healthcare infrastructure to improve informed dialysis choice and HoD rates.

Finally, we show a high congruence rates for those who select HoD (74%) and those who initiate HoD (62%). Drop-off of about 20% between choice and accrual have been shown by investigators from Europe and Asia. In a single available study from US, this difference was larger.[22] Our data did not allow for a comprehensive analysis of these drop-offs but through our anecdotal experiences we believe that the changes in social and medical factors drive these discrepancies. We further show that the choice of HoD is not impacted by the severity of CKD, a finding similar to our prior study. Together these findings allow and encourage early application of CPE for stage 4 CKD, but also provides evidence that CPE can be efficacious at any severity of CKD.

There are a few limitations with our study. First, one could argue as to why our protocol works as well as it does while others haven’t? However, this is not entirely true! Many international studies have shown effects comparable to ours, unfortunately this topic is not as well reported from within the US. Examining these international reports, largely from infrastructures with high HoD use, we presume that the availability of adequate HoD expertise may have allowed more patients to choose HoD in those infrastructures. Furthermore, heterogeneity in CPE format in the prior US reports may have impacted their outcomes data as there was limited information on the content, format and educator expertise in these reports. A well-defined, evidence-based CPE protocol with group and individual counseling session, which included our innovative lifestyle simulation discussions, and trained providers with expertise in patient education, may explain our high and consistent efficacy. Second, absence of a control group may lead to a perception that our patient population was somehow different. However, the study was conducted in a university practice with large proportion of Medicare/Medicaid coverage and minority representations. In addition, examining the effect against the historical trends, as well as it being comparable to our prior study, further emphasizes the true efficacy of our CPE protocol. We concede though that while we are able to comment on the efficacy of our concise CPE protocol, we cannot comment on the component of CPE most helpful for patients’ decision making since we did not examine this separately. Mann et al have shown that about half of those choosing HoD, do so after being provided the information in a printed and audio-visual format whereas, the remaining half do so after personal counseling.[26] Limitations in patient health literacy and need for individual patient centered counseling could explain these findings. Though subjectively we can validate these findings, formal assessments of three components of our CPE (group lesson, one-on-one counseling, and life-style simulations) are needed. Third, only 37% of our patients developed ESRD at the end of 18 months of follow up and the use data is limited to these individuals. Thus we cannot comment on the impact of CPE on the entire population. At the same time, ongoing growth in our HoD census and prevalence indicates an overall efficacy of the program. Finally, though the benefits of HoD use on health service utilization are well known, the impact of a resource consuming CPE program and its cost effectiveness is unknown. Based on the Medicare per patient per year cost difference in the range of $15–20,000, as well as our subjective assessments of resources, we strongly believe that implementing CPE may have substantial health service benefits including cost efficiency while delivering a more patient-centered care. The true cost-benefit ratio of these endeavors need further examinations.

To conclude, our current report in congruence with our recently published prior report, we show that a majority of US advanced CKD patients when provided with CPE, choose and use HoD. We also show that this selection and use is applicable to the entire spectrum of socio-economical status, and that CPE can overcome the disparities is HoD use. We also show that a universal CPE strategy consisting of group and individual counseling including lifestyle simulation discussions, can lead to rapid and substantial growth in HoD utilizations. Finally we show that our protocol is portable and that application of our protocol within any medium to large size healthcare institute can substantially improve informed HoD rates. Due to its benefits on patient reported and health services outcomes, improving informed HoD use has been a national goal for over two decades. Wider application of such validated protocols can significantly impact HoD rates in the community with potentials for overall improvements in HR-QoL and healthcare utilizations for a much wider population.

## Supporting information

S1 File UFL CPE protocol. (DOCX)
