# Supplementary material for: What do the US advanced kidney disease patients want? Comprehensive pre-ESRD Patient Education (CPE) and choice of dialysis modality
Source: PLoS One. 2019 Apr 9;14(4):e0215091. doi: 10.1371/journal.pone.0215091 (PMC6456188; doi:10.1371/journal.pone.0215091)
Supplement: S1 File — (DOCX) [file pone.0215091.s001.docx]

University of Florida Comprehensive pre-ESRD Patient Education Protocol:

**Eligibility for patients:**

All patients with established Chronic Kidney Disease of Stage 4 and 5 severity and not on dialysis.

For the purposes of this study, patients with special considerations including cognitive dysfunction, those living in assisted living facilities or in nursing homes, and those with life-expectancy of less than 6 months were excluded.

**Educators:**

Only provider trained the nephrology and dialysis care (content matter experts) can provide the CPE. The educator for the CPE shall be one of the following:

- A Renal Physician
- A Renal Physician Assistant
- A Renal Advanced Nurse Practitioner
- A Renal Nurse proficient in both in-center dialysis and home dialysis

New educator providing CPE requires on-the-job-training for the study protocol under the guidance of Drs. Shukla, prior to being instituted for the patient education role. New educator training includes a minimum of 3 sessions of training under the principles of observe, learn and adapt and assessments and feedbacks.

**CPE Syllabus:**

The outline of CPE has been sourced from the general guidelines established by NKF and CMS however, and all patients are encouraged to research freely available online resources from national kidney disease advocacy groups including: National Kidney Foundation (NKF) and Renal Physician Association (RPA), National Kidney Disease Education Program (NKDEP), Center for Medicare and Medicaid Services (CMS) and Veteran Healthcare Administration (VHA). However, key limitations of our education program are acknowledged.

- Knowledge of kidney disease is vast and many time overwhelming for most kidney disease patients.
- Though our CPE program is based on these recommendations and it has been organized in a manner that allows advanced CKD patients to learn about the kidney disease and its management options.
- Kidney Disease Education is provided under the following domains (Additional details can be obtained by contacting UF CKD education clinic, Gainesville, FL:
  - Basic structure and function of kidney
  - What is CKD and ESRD, understand the stages
  - Signs and symptoms of CKD
  - Common medical problems associated with CKD
  - Management options of ESRD including hands on demonstrations, and when to initiate therapy
  - Who pays for ESRD, disabilities and social needs
  - Fears, concerns, and frequently asked questions
- Finally, our CPE specifically focuses on the patients ability to understand the kidney disease with reference to their own life-style and preferences

**Take-home messages:**

- Based on well-published available data, all patients with advanced CKD should explore renal transplantation if feasible.
  - ‘If eligible, transplantation is the best modality of renal replacement therapy
- For those ineligible for transplantation, the cause of ineligibility for transplantation must be identified and advised correction.
- All patients with MDRD eGFR below 20ml/min should be encouraged to for the live-donor renal transplantation evaluation if feasible, and enlist for the deceased donor transplantation listing
- The following dialysis related messages must be unequivocally conveyed during pre-ESRD education. Each of these statements must be confirmed affirmative by the patient at the end of education session.
  - The education must be provided without bias towards any particular dialysis modalities
  - All dialysis modalities are equivalent in terms of efficacy, i.e. provide equivalent medical outcomes, such as survival
  - Dialysis modality choice is a matter of patient preference
  - Though one might have apprehension, all attempts must be made to ensure that the fear is not an overriding motivator in dialysis choice
  - Dialysis modality should be chosen based on the life-style considerations

**CPE Format:**

All patients referred to the CPE for the first time should undergo the new patient protocol which will include group education, face-to-face education and life-style simulation discussions. All returning patients should be encouraged to attend the group education, but the group education will be optional for the returning patients and will be allowed to attend direct face-to-face session

The face-to-face counseling should follow a protocol based discussion designed to

- Ensure the group session comprehension- if needed review the content
- Understand home living situation, availability of social support structure, and their life-style preferences
- Assess the medical comorbidities driving the modality choice
- Review the Frequently Asked Questions.

The life-style simulation discussions will follow a generic weekly schedule for the patient in their current and desired life-style

- The modality choice will be discussed in an active manner; ensuring that it reflects the patient’s choice and not fears.
- The modality choice will be recorded in a passive manner; ensuring full patient autonomy
